# Farming Systems, Food Security, Dietary Intakes, and Nutrition Status Among Young Children in Rural Tanzania Before and After Harvest

**DOI:** 10.1111/mcn.70178

**Published:** 2026-06-07

**Authors:** Happiness Muhimbula, Joyce Kinabo, Aifric M. O'Sullivan

**Affiliations:** ^1^ UCD Institute of Food and Health, UCD School of Agriculture and Food Science University College Dublin, Belfield Dublin Ireland; ^2^ Department of Human Nutrition and Consumer Sciences Sokoine University of Agriculture Morogoro Tanzania

**Keywords:** agriculture, child nutrition, farming system, food security, infant nutrition, nutrition status, stunting, undernutrition

## Abstract

Subsistence farming households are at risk of food insecurity and poor nutritional status due to dependence on farm production for consumption. This study aimed to examine the relationship between household farming systems, food security and infant nutrition status pre‐ and post‐harvest. Households with mothers and infants and young children < 24 months were recruited from rural villages in Morogoro and Shinyanga, Tanzania. Demographics, anthropometrics, dietary intakes and household food insecurity were recorded. ANCOVA was used to examine differences and interactions between farming systems, seasons, food security, and nutrition status. The results showed high food insecurity pre‐harvest, highest within the Mixed Crop Livestock (MCL) farming system (82%) and Mixed Food Crop (MFC) farming system (74%). Post‐harvest, food insecurity was lowest for MCL households (21%) and highest for Single Food Crop (SFC) households (49%). MFC infants and young children had lower WAZ and LAZ and Cash Crop (CC) infants and young children had higher WAZ and LAZ pre‐harvest. Within the MFC and MCL farming systems, a significant increase pre to postharvest in MUAC was observed (*p* < 0.01 and *p* < 0.001, respectively). This study highlighted high food insecurity, poor dietary intakes and poor nutrition status pre‐harvest. Our findings revealed that the CC farming system had a greater income‐generating pathway and that MCL and MFC families had food group diversification post‐harvest. Therefore, interventions to improve nutrition status should improve both farming systems, while addressing seasonal gaps through food storage and preservation, market access and nutrition education for better child nutrition.

## Summary

- This study highlighted high food insecurity, poor dietary intake, and poor nutrition status in rural farming households in Tanzania pre‐harvest.
- Infants and young children from different farming systems had different risks of undernutrition.
- The proportion of households classified as food secure increase post‐harvest with differences between farming systems.
- The proportion of infants and young children classified as stunted, wasted, or underweight decreased post‐harvest, with differences between farming systems.
- The changes in food security, food intake, and nutrition status post‐harvest indicate that agriculture has the potential of increasing food access and nutrition status.

## Graphical abstract

Harvest‐season has a significant impact on food security and dietary intakes which in turn affects infant and young child nutrition outcomes in Tanzanian farming households. This research highlights the need for programmes and policies that will support households to adopt more targeted nutrition sensitive farming practices.

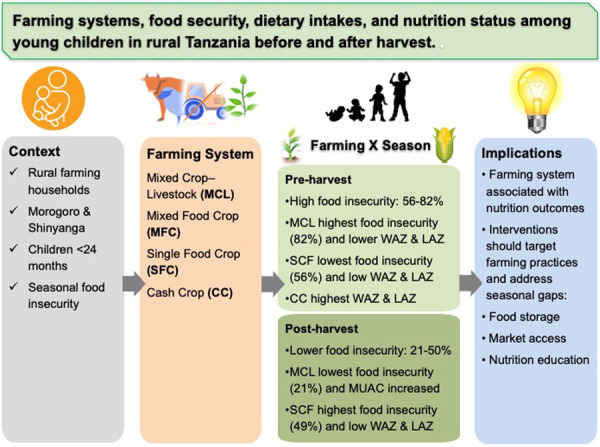

## 1 Introduction

Undernutrition during early life is a major global concern. Undernutrition is common in subsistence‐farming households were farming practices and seasons impact nutrition status through effects on food consumption. The UNICEF conceptual framework describes the immediate, underlying, and basic causes of malnutrition (Moursi et al. 2008). There are several examples of nutrition‐specific interventions that target immediate causes of undernutrition such as maternal and child food intake, promoting appropriate infant feeding, and treating infectious diseases (Arsenault et al. 2014). However, focusing on the immediate causes of undernutrition is not enough. Nutrition‐sensitive programmes addressing underlying determinants such as household food insecurity, inadequate prenatal care for women, and unhealthy living environments are essential for sustainable improvements in nutrition (Hotz and Gibson 2007). The Food and Agriculture Organisation (FAO) defines food insecurity as a situation that exists when people lack secure access to sufficient amounts of safe and nutritious food for normal growth and development and active and healthy life (Masset et al. 2011). Studies show that children living in food insecure households have low diet diversity and are at higher risk of undernutrition (Ali et al. 2019; Chandrasekhar et al. 2017; Haddad 2013). Rural food insecure households are often smallholder farming households that rely on their own production as their main source of food. Therefore, subsistence‐level farming is a key target for improving food security and potentially impacting nutrition status (Darapheak et al. 2013; Faber et al. 2016; Melese 2013). Several high‐level discussion documents have noted the potential for agriculture interventions to improve food security and nutrition outcomes in rural low‐income settings (Dewey 2001; Disha et al. 2012; Rodríguez et al. 2007). While interventions targeting farming practices have positive changes in infant feeding practices, dietary diversity, and food security (Dutta and Sharma 2020) very few report significant effects on nutrition status outcomes like stunting, wasting and underweight (Leonard et al. 2000; Michaelsen et al. 2017).

In farming households, time of year with respect to the growing season will impact food availability and access to food and therefore food security status, diet diversity and consequently nutrition status. However, very few studies have reported changes in food security status and food intake for farming households before and after harvest. Better knowledge of these relationships could help shape tailored nutrition‐sensitive interventions for households within different farming systems and at different times of the year. The current study examined the relationship between farming systems, food security, dietary intakes and nutrition status among infants and young children pre and post‐harvest seasons.

## 2 Methods

### 2.1 Study Design, Population and Sampling

This was a longitudinal survey design such that data were collected pre‐harvest and post‐ harvest. The study was conducted in the Mvomero district in the Morogoro region and the Kishapu district in the Shinyanga region, Tanzania Mainland. The regions were selected based on household farming practices representative of Tanzanian agriculture. The main crops in both regions include maize, rice, sorghum, sweet potatoes, green gram and groundnuts. Agriculture in both regions is dependent on season and rainfall, which follows a unimodal or bimodal rainfall pattern. Districts (within regions) are divided into divisions, and divisions are further sub‐divided into wards and villages. One division was randomly selected from each district, followed by the random selection of two wards per division, and one village per ward. The study villages were Makuyu and Milama in Mvomero district, and Lubaga and Mwakipoya in Kishapu district. The study population comprised of farming households with mothers and children < 2 years of age (nutrition status survey) randomly invited to the study from a larger study cohort, referred to here as the ‘general household survey’. Sample size calculations were performed using method described by the United Nations, powered at 90% with a type 1 error rate of 0.05. A sample of 277 households was recruited to the general household survey from each district (*n* = 554 households in total) based on estimates of food insecurity prevalence. A sample of 110 households from each district was recruited to the nutrition status survey based on estimates of wasting prevalence, including 10% attrition rate to mitigate potential losses to follow‐up post‐harvest. Pre‐harvest, considered a period of food shortage, was February‐March, 2014 and post‐harvest, considered a period of food surplus, was August, 2014.

### 2.2 Ethical Approval

This study was conducted according to the guidelines laid down in the Declaration of Helsinki, and all procedures involving human subjects/patients were approved by the National Institute of Medical Research (NIMR), Tanzania (Reference: 1679). Verbal informed consent was obtained from all subjects/patients. Verbal consent was witnessed and formally recorded. All data was collected by a team of trained researchers following standard protocols.

### 2.3 Demographic Information

A pre‐tested structured questionnaire was used to record information on age, sex and marital status of the head of the household, family size, headship of household, education, employment status, income, infant birth weight (reported on clinic card) and illness over the past 2 weeks (Muhimbula et al. 2019).

### 2.4 Identifying Farming Systems Within Farm Households

Farming systems were defined by researchers based on the types of crops cultivated, the degree of dependence and market orientation for crops, and the number of livestock units owned by households (Wordofa and Sassi 2020). The degree of household dependence on a particular crop was calculated based on the percentage of total land area cultivated that was allocated to that crop. The household was considered dependent on a particular crop if it had at least 70% of the total cultivated land area allocated to that particular crop. The placement of households into a specific farming system was based on the 2013 farming year data reported from the pre‐harvest survey (Wordofa and Sassi 2020). The farming systems were Single Food Crop (SFC) which grows a SFC on ≥ 70% of the total cultivated land; Mixed Food Crop (MFC) which grows several food crops, (i) none occupy ≥ 70% of the total cultivated land, and (ii) the total proportion of land dedicated to food crops is ≥ 70%; Cash Crop (CC) which grows a cash crop on ≥ 70% of the total cultivated land; and Mixed Crop Livestock (MCL) which grows crops and has more than 7.3 Tropical Livestock Units (TLU) (Wordofa and Sassi 2020).

### 2.5 The Household Food Insecurity Access Scale (HFIAS)

Household food insecurity was assessed using the Household Food Insecurity Access Scale (HFIAS) developed by the Food and Nutrition Technical Assistance (FANTA) project (Coates et al. 2007). The HFIAS tool consists of nine questions that capture all three core domains that reflect a household's inadequate access to food in the preceding 30 days. The response was recorded as 0 if food insecurity did not happen, 1 if it rarely happened (1–2 times), 2 if it sometimes happened (3–10 times) and 3 if it often happened (> 10 times) in the previous 30 days. Answers to questions were combined, and the household was allocated to a food security category as follows: food secure, mild food insecure, moderate food insecure and severely food insecure.

### 2.6 Anthropometry

Weight was measured using a seca weighing scales model 874 (seca Hamburg, Germany). Length and height were measured using the UNICEF portable length and height measuring system SET‐2 (S0114540). All anthropometric equipment was calibrated prior to use, and measurements followed standard protocols (NBS and ICF Macro 2015–16). Length‐for‐age z‐score (LAZ), weight‐for‐age z‐score (WAZ), and weight for‐length z‐score (WLZ) were calculated using WHO ANTHRO Software version 3.2.2 (WHO, Geneva, Switzerland) and WHO Child Growth Standards (De Onis 2006). Stunting, underweight and wasting were defined as z‐scores below −2 standard deviations (SD) of the median values of the reference data. Mid upper arm circumference (MUAC) was recorded using a flexible colour‐coded measuring tape (UNICEF, MUAC Child 11.5 Red/PAC‐50). The MUAC tape was wrapped around the mid‐upper left arm to measure its circumference, and measurements were taken to the nearest 0.1 cm. Based on the WHO/UNICEF recommended cut‐off points, children with MUAC ≤ 11.5 cm were classified as severely malnourished 11.5–12.5 cm as moderately malnourished and ≥ 12.5 cm as normal (WHO and UNICEF 2009).

### 2.7 Dietary Intake and Food Quantification

Individual semi‐quantitative 24 h dietary recalls were conducted pre and post‐harvest to determine the amount and types of foods consumed by children. Mothers recalled and described every item of food and drink the child had consumed over the past 24 h for 1 day only. Detailed information was obtained through systematic repetition of open‐ended questions. Amounts were estimated using standard household measures e.g. bowls, plates, cups, spoons. Where possible a measuring cylinder was used to estimate volumes or weights of foods consumed (Ferguson et al. 1994). All mixed dishes were probed for the type and amounts of ingredients used. All the information was recorded on the 24 h recall sheet. The 24 h recall was conducted on weekdays. Atypical days (religious feast or celebrations) were not included. The 24 h recall data was entered into NUTRITICS software research edition version 4.2 (Nutritics, Dublin Ireland), which included data from the Tanzanian Food Composition Database (Lukmanji et al. 2008). All foods consumed were categorised into 18 food groups defined using the nutrient composition of the foods and the Tanzania Food Composition database categories Table S1. Individual Dietary Diversity Scores for infants (IDDS) were also calculated based on WHO guidelines (WHO 2008). All foods that were consumed in the past 24 h were grouped into 7 food groups. Each group scored 1 point, otherwise 0. The IDDS scores were calculated by summing the number of food groups consumed by the individual children over the 24 h recall period. A score of ≥ 5 indicated minimum dietary diversity. Outliers (over‐or under‐reporters) for energy intake (EI) were identified prior to data analysis using a defined protocol as follows: (1) we calculated age dependent energy intake (EI)/energy requirements (ER) from complementary foods based on WHO criteria for breastfed children (Dewey and Brown 2003); (2) the ratio of EI/ER from complementary foods was determined for each child; (3) infants and young children with less than 50% of EI/ER or more than 150% EI/ER were identified as ‘*at risk of misreporting*’; (4) WAZ was examined for those ‘*at risk of misreporting*’; (5) food security status, breastfeeding frequency and infections for infants and young children identified as ‘*at risk of misreporting*’ were also examined. Over‐reporters were defined as those with WAZ < −2 and EI/ER > 150% coming from insecure households with no infections/illness in the months preceding the survey. Under‐reporters were defined as those with EI/ER < 50% from food‐secure households, with no reported illnesses. Based on the evaluation of data described in this protocol, food intake data from 3 children pre‐harvest classified as ‘*at risk of underreporting*’ were excluded from the analysis.

### 2.8 Statistical Analysis

A study database was compiled and imported into IBM SPSS Statistics for Windows for analysis version 20.0 (IBM Corp, NY, USA). Descriptive statistics were generated for all data, and normal distribution of continuous variables was checked graphically using box plot, Q‐Q plots and *p*‐values from the Shapiro–Wilk test. Non‐parametric tests were used when the data were not normally distributed. The findings were summarised using frequencies and proportions for categorical variables and mean and SD or median with interquartile ranges (IQR) for continuous variables. Where necessary, adjusted means and standard error of the mean (SEM) are presented. Baseline characteristics were compared for each of the farming systems using Chi‐square test for proportions and one‐way ANOVA with post‐hoc test to compare the means for continuous variables. Kruskal‐Wallis's test with post‐hoc ranking was used to compare the median amount of food consumed from food groups between the farming systems; the Mann‐Whitney Test was used to compare the amount of food consumed from food groups between food security status groups, and the Friedman test was used to examine the significant changes in the amount of food consumed from food groups pre‐ to post‐harvest. Univariate GLM ANCOVA was used to examine differences in WAZ, LAZ and WHZ between farming systems and food security status groups when pre‐ and post‐harvest data were examined separately. A two‐way mixed ANCOVA was used to test the effect of season on the changes in WAZ, LAZ and WHZ from pre‐ to post‐harvest and to examine interaction effects of farming system and food security on changes. Where the interaction term was significant, post hoc analysis involved separating groups to examine intra‐group changes between pre and post‐harvest. For univariate analysis all data was included except for individual missing observations for certain variables, for example if a measurement could not be taken for any reason. For two‐way analysis participant data was included if measurements were available for both time points. Mother's age and BMI, and infant and young child age, sex and birthweight were considered as potential covariates. In all cases, covariate selection followed the methods proposed by Greenland (1989). Infant and young child age and sex were significant and were included as the covariates in the final model. *p*‐values < 0.05 were considered statistically significant.

## 3 Results

### 3.1 Farming Systems, Demographics, and Food Security Status

Each household (*n* = 209) was allocated to one of four farming systems; SFC (23%), Mixed Food Crops (28%), Cash Crops (22%) and Mixed Crop‐Livestock (27%). Thirteen households were lost to follow‐up leaving a cohort of 196 post‐harvest. Characteristics of households by farming systems pre‐harvest are presented in Table 1. Household size was significantly different between farming systems (*p* < 0.001). MCL households had the highest number of household members (*n* = 10) and the highest proportion achieving formal education (> 40%, *p* < 0.001). Mothers from SFC households had the highest BMI in both seasons. There was no difference in infant birth weight across farming systems. About 40% of households experienced severe food insecurity pre‐harvest, but this decreased to 4% post‐harvest. Household food security status was different between farming systems pre (*p* = 0.01) and post‐harvest (*p* = 0.03). Food insecurity was lowest for SFC households (56%) and highest for MCL households (82%) pre‐harvest. Post‐harvest, food insecurity prevalence was lowest for MCL households (21%) and highest for SFC households (49%) (Table 2).

**Table 1:** Household characteristics by farming system groups.

	SFC (*n* = 48)		MFC (*n* = 58)		CC (*n* = 46)		MCL (*n* = 57)		Total (*n* = 209)		
Household*s*	Mean/median	SD/IQR	Mean/median	SD/IQR	Mean/median	SD/IQR	Mean/median	SD/IQR	Mean/median	SD/IQR	*p*‐value
Number of people	5.7	2.5	6.2	2.5	5.8	2.3	10.6	4.7	7.2	3.8	< 0.001
Female‐headed (%)	14.6		24.6		21.7		16.4		19.4		0.53
Married (%)	85.4		82.7		78.2		82.1		82.2		0.46
Years of school	6.6	1.7	6.9	1.2	7.3	2.1	6.3	1.4	6.8	1.9	0.18
No school (%)	16.7		15.5		15.2		42.9		23.1		0.001
Income (TZS mill)	1.6	1.9	1.5	1.7	3.9	6.6	5.5	7.7	3.1	5.4	< 0.001
Mother											
No school (%)	18.8		15.8		17.4		19.6		17.9		0.69
Age	29	22–33	27	22–31	29	23–24	24	22–35	27	21–33	0.75
Pre‐BMI (kg/m2)	23	20–25	21	19–23	22	20–23	21	20–23	22	20–24	0.03
Post‐BMI (kg/m2)	23	20–26	22	20–24	22	20–24	22	20–24	22	20–25	0.35
Child											
Age (mean, SD)	10.3	5.5	11.8	5.1	10.3	5.6	10.1	5.6	10.7	5.0	0.30
Males (%)	45.8		55.2		56.5		47.4		49.5		0.61
Birth weight (kg)	3.4	0.5	3.3	0.4	3.3	0.5	3.2	0.4	3.3	0.5	0.28

*Note:* Values are percentages, mean (SD), or median (IQR), depending on the distribution of continuous variables. *p*‐values are based on ANOVA, Kruskal‐Wallis, or Chi‐Square tests where appropriate.

Abbreviations: CC, cash crops; MCL, mixed crop‐livestock; MFC, mixed food crops; SFC, single food crop.

**Table 2:** Food security status of each farming system group pre and post‐harvest.

	SFC (*n* = 48)	MFC (*n* = 58)	CC (*n* = 46)	MCL (*n* = 56)		
	%	%	%	%	*χ* ^2^	*p*‐value
Pre‐harvest food secure	43.8	25.9	39.1	17.9	10.3	0.01
Pre‐harvest food insecure	56.3	74.1	60.9	82.1		
Post‐harvest food secure	51.1	64.8	68.9	78.8	8.5	0.03
Post‐harvest food insecure	48.9	35.2	31.1	21.2		

*Note:* Values are percentages. Food security status is defined using the household food insecurity access scale. *p*‐values are based on the Chi‐Square test (*χ* ^2^).

Abbreviations: CC, cash crops; MCL, mixed crop‐livestock; MFC, mixed food crops; SFC, single food crop.

### 3.2 Farming Systems and Breastfeeding Practices

Some key breastfeeding indicators are presented in Table S1. There was no significant difference in the proportion of mothers who initiated breastfeeding within 1 h of birth across farming systems. Approximately 30%–50% of mothers offered extra fluids other than breast milk within 3 days after delivery; however, the proportion of mothers offering fluids other than breast milk was not significantly different across farming systems. Plain water was the most frequent fluid offered in the first 3 days after birth. Approximately 80%–85% of infants were not exclusively breastfed from 2 to 5 months, but there was no difference in the proportion of infants exclusively breastfed across farming systems.

### 3.3 Farming Systems, Food Security, and Nutrition Status

Table 3 presents mean infant nutrition status measurements grouped by farming system and food security status pre‐ and post‐harvest. During pre‐harvest, infant WAZ (*p* = 0.01) and LAZ (*p* = 0.03) were different across farming systems, however the differences were not significant post‐harvest. Post‐hoc analysis revealed lower WAZ and LAZ among infants and young children from MFC households and higher WAZ and LAZ from CC households pre‐harvest after controlling for age and sex. WAZ, LAZ, WLZ, and MUAC also differed by food security status (Table 3). Infants in food‐insecure households had significantly lower WAZ (*p* = 0.01), WLZ (*p* = 0.01), and MUAC (*p* = 0.03) pre‐harvest. Two‐way ANCOVA revealed a significant interaction between food security status and the farming system on LAZ in the post‐harvest season (*p* = 0.01). GLM profile plots indicated that infants and young children from food‐insecure MCL had significantly higher LAZ than infants and young children from food‐secure MCL, whereas the opposite was the case for all other farming systems. Figure S1 There was no significant interaction between food security status and farming systems for WAZ, WLZ, and MUAC pre and post‐harvest (data not shown). The proportion of infants classified as stunted, underweight, wasted, and malnourished with low MUAC stratified by farming systems are presented Table S2. The proportion of underweight infants was significantly higher in the post‐harvest within CC farming households compared to other farming households (*p* = 0.04). Stunting rate increased in all farming systems during post‐harvest, with a significant increase for infant's CC farming households (*p* < 0.001). Similarly, the proportion of malnourished infants within SFC, MFC and MCL was significantly lower post‐harvest (*p* > 0.001).

**Table 3:** Nutrition status indicators for farming system groups and food security status groups.

		Pre‐harvest									Post‐harvest							
		WAZ		LAZ		WLZ		MUAC			WAZ		LAZ		WLZ		MUAC	
	*N*	Mean	SEM	Mean	SEM	Mean	SEM	Mean	SEM	*N*	Mean	SEM	Mean	SEM	Mean	SEM	Mean	SEM
SFC	45	−0.75	0.17	−1.41	0.17	0.01	0.19	14.3	0.2	43	−0.56	0.18	−1.70	0.17	0.38	0.19	14.8	0.2
MFC	57	−1.07	0.15	−1.58	0.15	−0.24	0.17	14.2	0.2	53	−0.77	0.16	−1.87	0.17	0.18	0.17	14.9	0.2
CC	45	−0.32	0.17	−0.92	0.17	0.24	0.19	14.7	0.2	42	−0.33	0.18	−1.45	0.17	0.54	0.19	15.2	0.2
MCL	56	−0.60	0.15	−1.20	0.15	0.10	0.17	14.1	0.2	49	−0.56	0.17	−1.43	0.16	0.20	0.17	14.8	0.2
*p*‐value		0.01		0.03		0.31		0.21			0.38		0.16		0.46		0.34	
Food secure	61	−0.39	0.15	−1.28	0.15	0.40	0.16	14.6	0.2	119	−0.53	0.11	−1.51	0.10	0.28	0.11	15.0	0.1
Food insecure	142	−0.83	0.09	−1.30	0.09	−0.14	0.11	14.2	0.1	59	−0.68	0.16	−1.81	0.15	0.29	0.16	14.8	0.2
*p*‐value		0.01		0.91		0.01		0.03			0.62		0.80		0.17		0.48	

*Note:* Values are adjusted means (SEM). *p*‐values for between‐group comparisons within a season are presented in the table and are based on GLM ANCOVA controlling for infant and young child age and sex.

Abbreviations: CC, cash crops; MCL, mixed crop‐livestock; MFC, mixed food crops; SFC, single food crop.

The second stage of GLM analysis focused on changes from pre‐ to post‐harvest Table S3. When the cohort was considered as a whole, there was a significant change in MUAC pre‐ to post‐harvest (*p* < 0.001). When each farming system was considered separately, a significant increase in MUAC was noted for children within the MFC (*p* < 0.001) and MCL (*p* = 0.01) systems; however, there were no significant changes in any other parameters. When food secure and food insecure groups were considered separately, there was a significant change in LAZ (*p* = 0.01) and MUAC (*p* = 0.01): LAZ decreased significantly in food insecure households, while MUAC increased significantly in food secure and insecure households. Two‐way repeated measures ANCOVA showed no interaction between food security and the farming system on nutrition status indicators. However, when food security status was included as a factor in repeated measures analysis for each farming system, there was a significant difference in the change of WAZ for food secure versus food insecure children from MFC households (*p* = 0.04). GLM profile plots revealed a greater decrease in WAZ for the food insecure group in the MFC farming system compared to the food secure group. No other differences were noted for food‐secure versus food‐insecure groups within the different farming systems.

### 3.4 Food Security and Infant and Young Child Food Intake

Based on one 24 h recall, all children in the study consumed ‘Cereals dishes’, ‘Vegetables dishes’ was the next most commonly consumed food group (41% pre, 51% post), followed by ‘Pulses dishes’ (19% pre, 39% post). ‘Meat, poultry, eggs, and fish’ were consumed by 10% pre‐harvest and 29% post‐harvest, and ‘Milk and milk products’ were consumed by 11% pre‐harvest and 13% post‐harvest. No child consumed foods from the ‘Vegetables’ or ‘Sugars’ groups pre‐harvest, only 4 children consumed the sugars group post‐harvest. There were no significant differences in the proportion of consumers from food‐secure and food‐insecure households; however, there was a significant difference in the amounts of foods consumed pre‐harvest (Table 4). Infants and young children from food‐insecure households had higher intakes of ‘Vegetable dishes’ (*p* = 0.04) and lower intakes of ‘Fruits and fruit juices’ compared to those from food‐secure households (*p* = 0.02). The % consumers of food groups from food insecure and food secure households increased post‐harvest except for ‘Pulses dishes and Milk and milk products’. While acknowledging the fact that children were 6 months older post‐harvest and they would consume greater amounts of foods, the median amount of ‘Cereal dishes’ consumed by 12–18 month old children increased from 242 g pre‐harvest to 297 g post‐harvest. Changes in the median amount of food consumed from each food group pre to post‐harvest were also compared for food‐secure and food‐insecure households separately depending on the available data at both time points. There was no significant difference in the amount of food consumed pre‐ to post‐harvest for any food group within food‐secure or food‐insecure groups.

**Table 4:** Percentage of consumers and the amount (g) of food consumed by children aged 6–24 months by food security status groups pre‐ and post‐harvest.

	Pre‐harvest							Post‐harvest						
	Food secure (*n* = 50)			Food insecure (*n* = 114)				Food secure (*n *= 118)			Food insecure (*n *= 58)			
	%	Median (g)	IQR (g)	%	Median (g)	IQR (g)	*p*‐value	%	Median (g)	IQR (g)	%	Median (g)	IQR (g)	*p*‐value
Cereals dishes	100	233	184–300	100	287	170–424	0.07	100	303	224–423	100	299	210–398	0.86
Cereals products	10	32	48–0	1.8	14	—	0.05	6.8	16	16.5–0	6.9	29.5	37.3	0.55
Vegetable dishes	32.8	51	43–79	46.5	88	43–121	0.04	39.1	102	44–212	57.6	51	44–104	0.14
Vegetables	—	—	—	—	—	—	—	—	—	—	1.7	—	—	
Pulses, nuts and seeds	10.9	10	5–16	15.3	9	5–18	0.78	3.1	9.5	4–17	23.6	10	5–13	0.97
Pulses, nuts and seeds dishes	25.0	38	21–97	16.0	76	38–101	0.33	45.5	89	50–102	36.1	54	36–101	0.37
Roots tubers banana dishes	4.7	54	36–0	4.9	88	75–150	0.18	7.8	132	75–162	18.1	123	56–171	0.86
Roots tubers banana	1.7	—	—	—	—	—	—	—	—	—	—	—	—	—
Milk and milk products	14.1	150	120–400	12.5	150	79–205	0.45	14.1	146	94–196	9.0	81	25–205	0.35
Meat poultry eggs fish	14.1	42	25–42	8.3	69	28–100	0.26	25.0	79	36–88	31.3	65	36–118	0.91
Fruits and fruit juices	12.5	50	31–87	13.2	5	5–50	0.02	17.2	37.5	12–100	11.8	77	33–100	0.36
Local broth	10.9	45	44–101	18.8	51	20–101	0.57	—	125	50–0	2.8	31	23–0	0.33
Sugar sweetened beverages	12	200	225–0	3.5	135	120–0	0.66	3.4	150	150–0	1.7	—	—	0.15
Teas	20.3	150	100–200	11.1	100	55–150	0.15	21.9	135	75–200	16.0	137	90–200	0.82
Sugars	0	—	—	0	—	—	—	2.5	10	—	1.7	—	—	—
Oils and fat	6	4.9	—	2.6	7.7	—	0.82	2.5	11.4	—	3.4	5.8	—	0.08
DDS (Mean, SD)		2.65	1.01		2.67	0.91	0.92		3.1	0.71		3.1	0.7	0.66

*Note:* Food intake data is based on a single 24 h recall. Values are percentages (%) consumers and the median (IQR) amount of food (g) consumed. *p*‐values for between‐group comparisons of the amount of food (g) consumed within a season are presented in the table and are based on Mann–Whitney tests. There was no significant difference in % consumers between groups within a season based on the Chi‐Square test (*χ* ^2^) (*p*‐values not presented). *p*‐values for changes pre‐ to post‐harvest are not presented in the table. Note. Cereals dishes, Root, Tubers and Banana dishes, Pulses, nuts and seeds dishes and Vegetables dishes presented here are the cooked dishes or relish according to the Tanzania food composition tables.

### 3.5 Farming Systems and Infant and Young Child Food Intake

The three most commonly consumed food groups for SFC and MFC farming systems were ‘Cereals dishes’, ‘Pulses dishes’, and ‘Vegetable dishes’. ‘Cereals dishes’ also ranked highest for CC, followed by ‘Vegetable dishes’ and ‘Local broths’. Again, ‘Cereals dishes’ and ‘Vegetables dishes’ were highest for MCL, followed by ‘Milk and milk products’. Food group intake by farming systems during pre‐harvest is presented in Table 5. There was a significant difference in the % consumers of ‘Vegetables dishes’ (*p* = 0.02) and ‘Pulses dishes’ (*p* = 0.02) across farming systems. More infants and young children in MFC and MCL farming systems consumed ‘Vegetables dishes’ compared to SFC and CC. SFC were the largest consumers of ‘Pulses dishes’. When comparing the amounts of food from food groups consumed, MCL had higher intakes of ‘Pulses dishes’ (*p* = 0.01) compared to all other farming systems.

**Table 5:** Percentage consumers and the amount (g) of food consumed by children aged 6–24 months by farming systems groups pre‐harvest.

	SFC (*n* = 37)			MFC (*n *= 49)			CC (*n* = 36)			MCL (*n* = 42)				
Farming system	%	Median (g)	IQR (g)	%	Median (g)	IQR (g)	%	Median (g)	IQ (g)	%	Median (g)	IQR (g)	*P*†	*P‡*
Cereals dishes	100	250	154–448	100	272	177–373	100	262	200–373	100	274	148–396	0.95	0.31
Cereals products	4.2	—	—	1.7	—	—	2.2	60	20–	1.8	30	12	—	—
Vegetable dishes	27.1	55	44–91	53.4	88	44–106	28.3	51	22–69	54.4	80	44–121	0.25	0.02
Vegetables	—	—	—	—	—	—		—	—	—	—	—	—	—
Pulses, nuts and seeds	6.3	12	9–21	12.1	10	5–18	10.9	4.9	2–30	24.6	8	5–15	0.62	0.02
Pulses, nuts and seeds dishes	27.1	38	22–101	22.4	38	38–88	19.6	38	19–93	7.0	82	62–136	0.01	0.02
Roots tubers and banana dishes	4.2	93	36–150	3.4	87	75–100	8.7	82	65–287	3.5	56	24–56	0.83	0.55
Roots tubers and banana	—	—	—	—	—	—		—	—	—	—	—	—	—
Milk and milk products	4.2	375	150–	15.5	160	77–300	2.2	—	—	26.4	150	100–200	0.31	0.01
Meat, poultry, eggs fish	—	—	—	—	—	—	—	—	—	1.7	—	—	—	—
Meat, poultry, eggs fish dishes	12.5	49	40–61	6.9	55	25–100	10.9	44	30–126	10.5	43	27–81	0.96	0.68
Fruits and fruits juices	16.7	50	25–50	8.6	5	5–50	10.9	5	5–52	15.8	10	5–75	0.14	0.20
Local broth	14.6	60	44–102	13.8	51	33–85	21.7	44	20–101	15.8	30	20–97	0.61	0.69
Sugar‐sweetened beverage	8.3	160	105–207	0	—	—	8.7	175	75–237	3.5	300	100	0.92	*0.12*
Teas	18.8	100	50–175	13.8	100	77–150	17.4	100	56–156	7	175	150–237	0.21	—
Sugars	0	—	—	0	—	—	0	—	—	0	—	—	—	—
Oils and fats	2.1	9.6	7.9–	0	—	—	2.2	—	—	5.3	4.9	4–	0.12	0.28
DDS (Mean, SD)	—	2.5	1.01	—	2.1	0.94	—	2.5	1.14	—	2.8	1.08	0.17	—

*Note:* Food intake data is based on one 24 h recall. Values are percentages (%) consumers and the median (IQR) amount of food (g) consumed. Mean and standard deviation are presented for diet diversity score across farming systems (DDS). *P*† are the *p*‐values for between‐group comparisons of the amount of food (g) consumed within a season and are based on Kruskal‐Wallis tests and one‐way ANOVA for DDS. *P*‡ are the *p*‐values for between‐group comparisons of the % consumers within a season and are based on Chi‐Square tests (*χ* ^2^). – Indicates that the number of consumers was less than three (*n* < 3); therefore, median and interquartile range (IQR) were not calculated or presented. Cereal dishes, root and tubers, pulses, nuts and seeds dishes and vegetables dishes presented here are the cooked dishes or relish according to the Tanzania food composition table.

Abbreviations: CC, cash crops; MCL, mixed crop‐livestock; MFC, mixed food crops; SFC, single food crop.

During post‐harvest, there was no change in the 3 most commonly consumed food groups for SFC and MFC households Table 6. The proportion of children consuming ‘Local broths’ in the CC households decreased, so the 3rd most commonly consumed food group was ‘Pulses dishes’. The proportion of MCL children consuming Milk and milk products also decreased so that ‘Pulses dishes’ were the 3rd most commonly consumed food group in this farming system. There were significant differences in the %consumers of 5 food groups post‐harvest, with MCL having the highest %consumers of ‘Vegetables dishes’ (*p* = 0.01) and ‘Pulses dishes’ (*p* = 0.02), but the lowest %consumers of ‘Pulses’ (*p* < 0.001). In contrast, SFC had the highest %consumers of ‘Pulse dishes’ but the lowest %consumers of ‘Pulses dishes’ and ‘Vegetables dishes’. MFC and CC had somewhat similar patterns of food intake post‐harvest, with slightly higher % consumers of Meat poultry eggs fish food group in the CC farming system. When amounts of food consumed by groups were compared, MFC and MCL had higher intakes of ‘Vegetable dishes’ (*p* = 0.04), ‘Pulses dishes’ (*p* = 0.003), and Meat poultry eggs (*p* = 0.04) and lower intakes of ‘Fruit and fruit juices’ (*p* = 0.02) compared to SFC and CC. There was a significant difference in the amount of ‘Pulses dishes’ (*p* = 0.01) consumed with the highest intakes in MFC and lowest in MCL households.

**Table 6:** Percentage consumers and the amount (g) of food consumed by children aged 6–24 months by farming systems post‐harvest.

	SFC (*n* = 47)			MFC (*n* = 53)			CC (*n* = 42)			MCL (*n* = 51)				
Farming systems	%	Median (g)	IQR (g)	%	Median (g)	IQR (g)	%	Median (g)	IQR (g)	%	Median (g)	IQR (g)	*P*†	*P‡*
Cereals dishes	100	244	204–354	100	305	201–518	100	340	255–392	100	302	225–445	0.35	0.42
Cereals and cereal products	10.4	15	10–38	6.9	24	10–46	4.3	17	16–0	1.8	—	—	—	*—*
Vegetable dishes	35.4	51	42–103	56.9	99	21–151	45.7	45	30–102	66.7	102	51–142	0.04	0.01
Vegetables	2.1	—	—	—	—	—	—		—	—	—	—	—	—
Pulses, nuts, and seeds	10.4	12.5	8–17	10.3	14	4–31	13.0	12	5–21	33.3	9	5–12	0.01	0.02
Pulses nuts, and seeds dishes	52.1	54	36–108	44.8	89	46–101	45.7	51	36–101	15.8	101	72–102	0.003	< 0.001
Roots, tubers, and banana dishes	4.2	217	171–	15.5	150	75–160	10.9	78	75–85	26.3	132	80–171	0.24	0.012
Roots, tubers and banana	2.1	—	—	—	—	—	—	—	—	—	—	—	—	—
Milk and milk products	4.2	175	100–	10.3	146	33–192	0	—	—	24.6	137	74–196	0.81	0.001.
Meat poultry eggs fish	2.1	—	—	1.7	—	—	6.5	25	—	1.8	—	—	—	—
Meat poultry eggs fish dishes	25.0	43	36–108	20.7	78	19–81	27.8	53	36–88	26.3	81	43–162	0.04	0.03
Fruits and Fruit juices	14.6	77	76–100	17.2	26	11–100	10.9	127	50–150	10.5	9	5–44	0.02	0.68
Local broth	0	—	—	4	6.9	31–125	0	—	—	0	—	—	*—*	—
Sugar‐sweetened beverage	2.1	—	—	1.7	—	—	6.5	—	—	0	—	—	—	—
Teas	25	122	55–200	24.1	135	95–200	15.2	200	150–250	7.0	75	42–212	0.18	0.04
Sugars	0	—	—	1	—	—	0	—	—	3	9	—	—	—
Oils and fat	4.2	6	5.6–	0	—	—	4.3	17	11–	3.5	—	—	—	—
DDS (Mean, SD)	—	3.0	0.79	—	3.1	0.76		3.1	0.83	—	3.2	0.81	0.94	—

*Note:* Food intake data is based on one 24 h recall. Values are percentages (%) consumers and the median (IQR) amount of food (g) consumed from single‐day consumption. Mean and standard deviation are presented for the dietary diversity score across farming systems (DDS). *P*† are the *p*‐values for between‐group comparisons of the amount of food (g) consumed within a season and are based on Kruskal‐Wallis tests with post‐hoc ranking. One‐way ANOVA for DDS *P*‡ are the *p*‐values for between‐group comparisons of the % consumers within a season and are based on Chi‐Square tests (*χ* ^2^). – Indicates that the number of consumers was less than three (*n* < 3); therefore, median and interquartile range (IQR) were not calculated or presented. Cereals, Root and Tubers, Pulses, and Vegetables presented here are the cooked dishes or relish made from vegetables according to the Tanzania food composition table.

Abbreviations: CC, cash crops; MCL, mixed crop‐livestock; MFC, mixed food crops; SFC, single food crop.

Changes in median amount of food consumed from food group's pre to post‐harvest were compared by splitting the file by farming systems. The amounts of ‘Cereals dishes’ consumed increased significantly from pre to post‐harvest for MCL (*p* = 0.003) and SFC (*p* = 0.04) households. Children's intakes of ‘Vegetables dishes’ also increased significantly in SFC farming households (*p* = 0.04). Intakes of other food groups could not be compared given the number of valid cases pre and post.

## 4 Discussion

This study reports differences in food security and nutrition status outcomes for infants and young children from different farming systems pre and post‐harvest. Infants and young children from MFC households had lower mean WAZ and LAZ compared to other farming systems, while infants and young children from CC households had higher mean WAZ and LAZ. Infants and young children from food‐insecure households had lower WAZ, WLZ, and MUAC pre‐harvest, whereas there was no difference post‐harvest. Farming systems with a low prevalence of food security pre‐harvest improved post‐harvest; for example, the proportion of MFC and MCL households classified as food secure increased from 20% to 80%. Changes in food security coincided with changes in food intake and MUAC for MFC and MCL households. The SFC farming system was different from others in that the proportion of food‐secure households did not change much from pre‐ to post‐harvest. Overall, CC farming households recorded better WAZ, LAZ, WLZ and MUAC compared to other farming systems. While acknowledging the fact lower LAZ observed post‐harvest may not be directly related to higher nutritional risk observed pre‐harvest season due to influences on linear growth as infants were 6 months older post‐harvest; Improvements in nutrition status post‐harvest were linked to increased food security and food group consumption. When comparing diets across farming systems, there were no major differences in the proportions consumed or the amounts of foods consumed pre‐harvest. However, farming system‐related dietary patterns were more apparent post‐harvest.

Among all farming systems, MCL and MFC households were the most sensitive to seasonal changes in food accessibility, which was reflected in improved food security, improved food consumption and higher MUAC post‐harvest. Food group diversity was poor pre‐harvest for MCL and MFC households; both % consumers and the amount of food consumed increased post‐harvest, particularly for ‘Pulses, nuts and seeds’, ‘Pulses nuts and seeds dishes’ and Meat poultry eggs fish dishes. The difference between these farming systems is that MCL includes livestock. Research suggests that farming systems that mix crops and livestock can contribute to both farm income and food security with potential implications for improved nutrition status (Nabarro and Wannous 2014). In support of this finding, MCL households had better nutrition status compared to MFC who had lower WAZ, LAZ, and WLZ in both seasons. However, the high levels of food insecurity in MCL households pre‐harvest highlights the significant impact of season on food security and nutrition status regardless of farming practices, which is well documented in the literature (Ferro‐Luzzi et al. 2002; Harding 2008; Schalekamp 2009). From the general household survey (described in the methods), MCL households had high levels of food insecurity during pre‐harvest, had the biggest households, and had the highest age‐dependent ratio which is consistent with our findings (Massawe 2016). Using data collected from the households reported in this manuscript, as well as other households in both regions with children > 24 months, Massawe (2016) reported that MCL household income was generated from off‐farm activities mainly post‐harvest, suggesting poor access to cash and reserves for these households in the pre‐harvest season. Additionally, Massawe and his colleagues argued that although MCL households had the highest overall income, such income was seasonal, and it is possible that it was depleted in just a few months after the harvest period (Massawe 2016).

Similar high rates of food insecurity in pre‐harvest among livestock keepers were reported previously in Uganda (Mayanja et al. 2015). Although one might expect livestock owners to sell animals in times of food shortage for income and so that children can remain in school, this is typically not the case for households with livestock (Hedges et al. 2016). Research suggests that pastoralists prioritise livestock as they view livestock as a financial asset (Coppock et al. 2018), and therefore, children often leave school early to take care of livestock (Hedges et al. 2016). Therefore, income is not directly related to years of schooling in certain instances. Research suggests that this is not happening, possibly due to market access and the perceived impact on socioeconomic status (Ruhangawebare 2010; Stroebel et al. 2011). Additionally, from these findings, MCL households had the largest household sizes, while a large household size in MCL was important for providing labour for both livestock keeping and cropping activities, it could be associated with high food insecurity levels depending on their contribution to household income.

An unexpectedly significant interaction was observed between food insecurity and LAZ for infants and young children in MCL households. Infants and young children from food‐insecure MCL households had higher LAZ than food‐secure MCL post‐harvest. In contrast, all other systems showed better nutrition status in food‐secure than in food‐insecure households. It is difficult to know what mechanism might be at play here; however, the data shows that during the pre‐harvest season, a larger proportion of children from MCL households consumed ‘Milk and milk products’ compared to other households. At the same time, a small proportion of children were exclusively breastfed (~15%) and so a majority were receiving fluids and foods other than breastmilk. It is possible that a larger proportion of children in MCL households received milk as a substitute fluid during the pre‐harvest, food‐insecure period and that this led to somewhat better outcomes relative to other farming households. Whilst this is an extrapolation of the available data, we know from previous research that it is common to introduce animal milk to infants and young children in households with livestock in Tanzania (Hanselman et al. 2018; Kibona and Mwanri 2020). Several studies have shown a significant positive relationship between milk intake and linear growth (Ånestrand 2013; Hoddinott et al. 2015; Mosites et al. 2017; Shreenath et al. 2011; Wagah et al. 2015) & Haile and Headey (2023). Despite this observation and discussion around the potential that cow's milk was used as a substitute fluid for infants and young children in this period, it should be noted that the WHO/UNICEF recommend exclusive breastfeeding until 6 months, when suitable complementary foods are introduced alongside breastfeeding, with further advice to delay cow's milk until after 1 year of age (Victora et al. 2016).

Within the SFC system, the proportions of food secure and food insecure households did not vary much pre‐ and post‐harvest. Although food production diversity has potential to improve food security (Melese 2013; Sunderland 2011), mono‐cropping has the advantage of increasing yield (Weitzman 2000). However, in subsistence farming systems mono‐cropping is vulnerable to crop failure leading to higher food insecurity, poor diet quality and nutrition (Sunderland 2011). This might explain the observation that SFC households had higher food security in pre‐harvest compared to other farming systems, but very little change post‐harvest. Indeed, ~50% of SFC households were food insecure post‐harvest compared to other farming systems where the proportions of food insecure households dropped significantly. Food insecurity within SFC was related to poor nutrition status which recorded lower WAZ, LAZ and WLZ second to MFC for both pre and post‐harvest. Yet again, diet diversity was poor, reflected in the high intakes of ‘Cereal dishes’ and low intakes of ‘Pulses nuts and seeds’ ‘Milk and milk products and Meat, poultry, eggs, fish dishes’. SFC households also had low income relative to other farming systems and smaller land areas which probably explains their dependence on maize mono‐cropping for home consumption (Wordofa and Sassi 2020).

Agriculture generates income for food and non‐food expenditures, which can translate to expenditure on nutrition‐enhancing goods and services including health care and education and consequently impact child nutrition status (Gillespie and van den Bold 2017). Studies indicate that children in higher‐income households tend to eat a higher‐quality diet than those in poorer households (Arimond and Ruel 2004; Wiegers et al. 2011). However, other studies suggest that introducing cash crops in poorer households does not translate to improved nutrition status (Ecker et al. 2011). The current study showed that infants and young children from CC households had higher WAZ and LAZ compared to other farming systems. CC households had a relatively small proportion who had no schooling, were the farming system with the highest number of school years (although not statistically significant) and had higher income relative to SFC and MFC. The combination of these factors would suggest that CC households had some advantage over other households, which might result in better access to food, and health services, hence better nutrition status.

Food intakes were quantified based on groups defined in the Tanzanian Food Composition Database (Lukmanji et al. 2008). The amount and % consumers of food groups increased post‐harvest, and there were differences between farming systems, suggesting that dietary patterns differ when food is available. Several studies have reported similar findings in relation to production diversity and diet diversity (Ferguson et al. 2015; Hirvonen and Hoddinott 2016; Melese 2013; Organisation 2023). However, despite improved intakes post‐harvest, very few infants and young children consumed animal source foods, particularly meat, poultry, fish and eggs. This is concerning as low intakes of animal source foods are associated with nutrient deficiencies and increasing intakes are associated with improved growth and lower rates of stunting (Hetherington et al. 2017; Iannotti et al. 2017). The changes noted here when food is available suggests that an intervention that improves food production could have an impact on nutrition status. However, improving access to foods pre‐harvest remains a challenge. Multicomponent interventions that target food production, processing and preservation while also educating households and providing economic support systems could provide a more sustainable change and alleviate food shortages in the pre‐harvest season.

This study collected data from households in two regions of Tanzania selected as the farming practices broadly represented Tanzanian agriculture. The main strength of this study was the longitudinal study design which provided the means to link farming practices, food security, food consumption, and infant nutrition status while accounting for seasonal changes. However, the fact that children were 6 months older post‐harvest must be acknowledged when comparing food intakes pre‐ and post‐harvest as older children will consume greater amounts of foods. In this study, food records were quantified, and intakes were analysed from a food group perspective, which eases translation to practical recommendations. However, food intake was assessed based on one 24 h recall which has the potential to introduce recall bias and will not account for day‐to‐day variation and therefore is not a true reflection of habitual diet. Finally, the sample size in this survey was calculated to assess nutrition status; however, when split by farming system and given the low intakes of certain food groups it was not possible to calculate *p*‐values for certain comparisons which limited the analysis of food intake data.

## 5 Conclusion

This study revealed high food insecurity, poor diet diversity, and nutrition status pre‐harvest in four farming systems. While seasonal food insecurity affected all farming systems, children from CC households recorded better WAZ and LAZ than children from MFC and MCL households pre‐harvest. Food security and food intake increased post‐harvest, which was reflected in improved nutrition status, particularly for MFC and MCL households. This suggests that CC farming systems provide income that can support households during food shortages, while MFC and MCL systems improve dietary diversity post‐harvest. There were no major differences in dietary intake between the farming systems, particularly in pre‐harvest, when food availability was low for all. Dietary diversity only improved post‐harvest, particularly through increased consumption of legumes, pulses, nuts, and animal‐source foods.

Generally, our findings highlighted two important pathways: the income‐generating pathway, which was stronger in the CC farming system, and food group diversity, which was observed among MCL and MFC households post‐harvest. Interventions to improve nutrition status should therefore focus on strengthening both pathways while addressing seasonal gaps through improved food storage and preservation, enhancing market access for nutrient‐rich foods, and promoting nutrition education for better child nutrition.

## Author Contributions

H.M., J.K. and A.M.O.S. designed the study and carried out the study. H.M. and A.M.O. analysed the data and interpreted the findings. All authors contributed to writing the article.

## Conflicts of Interest

The authors declare no conflicts of interest.

## Supporting information


**Figure S1:** Interaction between food security and farming system on LAZ.

**Table S1:** Breastfeeding practices among children from farming households by farming systems. **Table S2:** The effect of farming systems and nutrition status of young children in agricultural households, controlling for age. **Table S3:** Changes in nutrition status pre to post‐harvest by farming systems and food security status.

## Data Availability

The data that support the findings of this study are available on request from the corresponding author. The data are not publicly available due to privacy or ethical restrictions.
