# Supplementary material for: Farming Systems, Food Security, Dietary Intakes, and Nutrition Status Among Young Children in Rural Tanzania Before and After Harvest
Source: Matern Child Nutr. 2026 Jun 7;22(3):e70178. doi: 10.1111/mcn.70178 (PMC13243768; doi:10.1111/mcn.70178)

**Supplementary Table 1: Breastfeeding practices among children from farming households by farming systems**

|  | **SFC** |  | **MFC** |  | **CC** |  | **MCL** |  | **P-values** |  |
| --- | --- | --- | --- | --- | --- | --- | --- | --- | --- | --- |
| Early Initiation of breastfeeding | **N** | **%** | **N** | **%** | **N** | **%** | **N** | **%** |  |  |
| Within 1 hour | 27 | 57.4 | 28 | 49.1 | 20 | 43.5 | 19 | 34.5 | 0.13 |  |
| After 1 hour | 20 | 42.6 | 29 | 50.9 | 26 | 56.5 | 36 | 65.5 |  |  |
| Total | 47 | 100 | 57 | 100 | 46 | 100 | 55 | 100 |  |  |
| Responses on feeding Fluids in 3 days after delivery |  |  |  |  |  |  |  |  |  |  |
| Yes | 14 | 29.2 | 30 | 51.7 | 17 | 37 | 21 | 37.5 | 0.14 |  |
| No | 34 | 70.8 | 28 | 48.3 | 29 | 63 | 35 | 62.5 |  |  |
| Total | 48 | 100 | 58 | 100 | 46 | 100 | 56 | 100 |  |  |
| Types of fluids given |  |  |  |  |  |  |  |  |  |  |
| Plain water | 12 | 66 | 14 | 41 | 8 | 38.3 | 14 | 56 | 0.24 |  |
| Sugar or glucose water | 3 | 17 | 5 | 15 | 6 | 28.5 | 3 | 12 |  |  |
| Cow's milk | - | - | 2 | 5.8 | 1 | 4.7 | 2 | 8 |  |  |
| Others (tea, herbal medicine, gripe water& orange juice) | 3 | 17 | 13 | 38 | 6 | 28.5 | 6 | 24 |  |  |
| Total | 18 | 100 | 34 | 100 | 21 | 100 | 25 | 100 |  |  |
| Feeding practices at 2-5 months |  |  |  |  |  |  |  |  |  |  |
| Only breast milk | 9 | 18.8 | 8 | 13.8 | 4 | 8.7 | 5 | 8.9 | 0.44 |  |
| A combination of breast milk with other fluids and solid foods | 38 | 79.2 | 49 | 84.5 | 39 | 84.8 | 48 | 85.7 |  |  |
| Total | 47 |  |  |  |  |  |  |  | |  |

SFC, Single Food Crop; MFC, Mixed Food Crops; CC, Cash Crops; MCL, Mixed Crop-Livestock. Values are percentages. P-values are based on the Chi-Square test (**χ2)**.

**Supplementary Table 2: The effect of farming systems and nutrition status of young children in agricultural households, controlling for age**

|  | **Farming systems attached to households** | | | | | | | |  | | |  |
| --- | --- | --- | --- | --- | --- | --- | --- | --- | --- | --- | --- | --- |
|  | **SFC(n=45)** | | **MFC (54)** | | **CC (n=44)** | | **MCL(n=52)** | | | **P-values** | | |
| **Nutrition status** | **Pre** | **post** | **Pre** | **post** | **pre** | **post** | **pre** | **post** | | pre | post | |
| % Underweight | 13.3 | 14.0 | 27.8 | 23.1 | 4.5 | 11.9* | 7.5 | 10.4 | | 0.003 | 0.04 | |
| % Stunting | 28.9 | 37.2 | 41.1 | 44.2 | 17.8 | 40.5** | 30.4 | 39.6 | | 0.91 | 0.36 | |
| % Wasting | 4.4 | 0.0 | 11.1 | 6.3 | 0.0 | 0.0 | 3.8 | 5.4 | | 0.08 | 0.38 | |
| % Malnourished | 13.3 | 2.3** | 10.7 | 1.9** | 7.0 | 2.4 | 11.1 | 6.3** | | 0.95 | 0.51 | |

***^a^*** Values are, mean with Standard deviation and percentages, P<0.05 for the differences in nutrition status across farming systems pre and post are derived from one-way ANOVA and Chi-square test. The significant differences within farming systems pre-and post-harvest were compared by a 2-sample T-test for continuous variables and the Mcnemar test for categorical variables. * Present significance at P<0.05 and ** at p<0.001

**Supplementary Table 3: Changes in nutrition status pre to post-harvest by farming systems and food security status**

|  | **N** | **WAZ** | **(178)** |  | **LAZ** | **(188)** |  | **WLZ** | **(178)** |  | **Hb** | **(183)** |  | **MUAC** | **(181)** |  |
| --- | --- | --- | --- | --- | --- | --- | --- | --- | --- | --- | --- | --- | --- | --- | --- | --- |
| **Variable** |  | **Pre** | **Post** | **P** | **Pre** | **Post** | **P** | **Pre** | **Post** | **P** | **Pre** | **Post** | **P** | **Pre** | **Post** | **P** |
|  | 188 | -0.67±0.08 | -0.57±0.74 | 0.41 | -1.29±0.08 | -1.65±0.08 | 0.09 | 0.05±0.09 | 0.30±0.08 | 0.39 | 9.5±0.1 | 9.4±0.1 | 0.02 | 14.4±0.1 | 14.9±0.1 | <0.01 |
| SFC | 41 | -0.75±0.17 | -0.56±0.18 | 0.58 | -1.41±0.17 | -1.70±0.17 | 0.87 | 0.01±0.19 | 0.38±0.19 | 0.88 | 9.6±0.2 | 9.4±0.2 | 0.66 | 14.1±0.2 | 14.9±0.2 | 0.65 |
| MFC | 50 | -1.07±0.16 | -0.77±0.16 | 0.95 | -1.60±0.15 | -1.87±0.15 | 0.17 | -0.24±0.17 | 0.18±0.17 | 0.75 | 9.4±0.2 | 9.4±0.2 | 0.73 | 14.2±0.2 | 14.9±0.2 | <0.01 |
| CC | 41 | -0.32±0.17 | -0.33±0.18 | 0.24 | -0.95±0.17 | -1.45±0.17 | 0.35 | 0.24±0.19 | 0.54±0.19 | 0.14 | 9.5±0.2 | 9.2±0.2 | 0.12 | 14.7±0.2 | 15.3±0.2 | 0.62 |
| MCL | 46 | -0.60±0.16 | -0.56±0.17 | 0.58 | -1.20±0.15 | -1.43±0.16 | 0.56 | 0.10±0.17 | 0.24±0.17 | 0.88 | 9.6±0.2 | 9.8±0.2 | 0.55 | 14.1±0.2 | 14.8±0.2 | 0.01 |
| Sec | 51 | -0.39±0.15 | -0.53±0.11 | 0.22 | -1.28±0.15 | -1.59±1.21 | 0.15 | 0.40±0.17 | 0.28±0.11 | 0.78 | 9.5±0.2 | 9.6±0.1 | 0.07 | 14.6±0.2 | 15.1±0.1 | 0.05 |
| Insec | 126 | -0.83±0.09 | -0.68±0.16 | 0.98 | -1.31±0.09 | -1.81±1.09 | 0.01 | -0.14±0.11 | 0.29±0.16 | 0.18 | 9.4±0.1 | 9.1±0.2 | 0.02 | 14.2±0.1 | 14.9±0.2 | 0.01 |

^a^Values are adjusted means ± SEM, P-values for changes pre to post-harvest are based on GLM repeated measures ANCOVA controlling for age and sex only; other variables were not statistically significant. Data are presented in the sequence of analysis: Split file by FSys (no between-subject factor); Split file by FSec (no between-subject factor). A repeated measures GLM was used for infants, with data from both pre- and post-harvest periods. P-values <0.05 were considered significant. SFC =Single Food Crop; MFC= Mixed Food Crops’=Cash Crops; MCL=Mixed Crop-Livestock; Sec= Food secure; Insec=Food Insecure

**Supplementary Figure 1: Interaction between food security and farming system on LAZ**


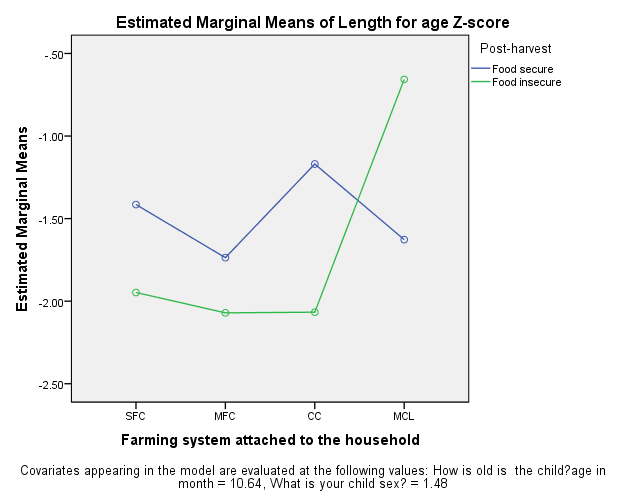

Supplement: Supplementary file 1 — Figure S1: Interaction between food security and farming system on LAZ. [file MCN-22-e70178-s001.docx]
