# Supplementary material for: Farming Systems, Food Security, Dietary Intakes, and Nutrition Status Among Young Children in Rural Tanzania Before and After Harvest
Source: Matern Child Nutr. 2026 Jun 7;22(3):e70178. doi: 10.1111/mcn.70178 (PMC13243768; doi:10.1111/mcn.70178)
Supplement: Supplementary file 2 — Table S1: Breastfeeding practices among children from farming households by farming systems. Table S2: The effect of farming systems and nutrition status of young children in agricultural households, controlling for age. Table S3: Changes in nutrition status pre to post‐harvest by farming systems and food security status. [file MCN-22-e70178-s002.pdf]

Supplementary Figure 1: Interaction between food security and farming system on LAZ

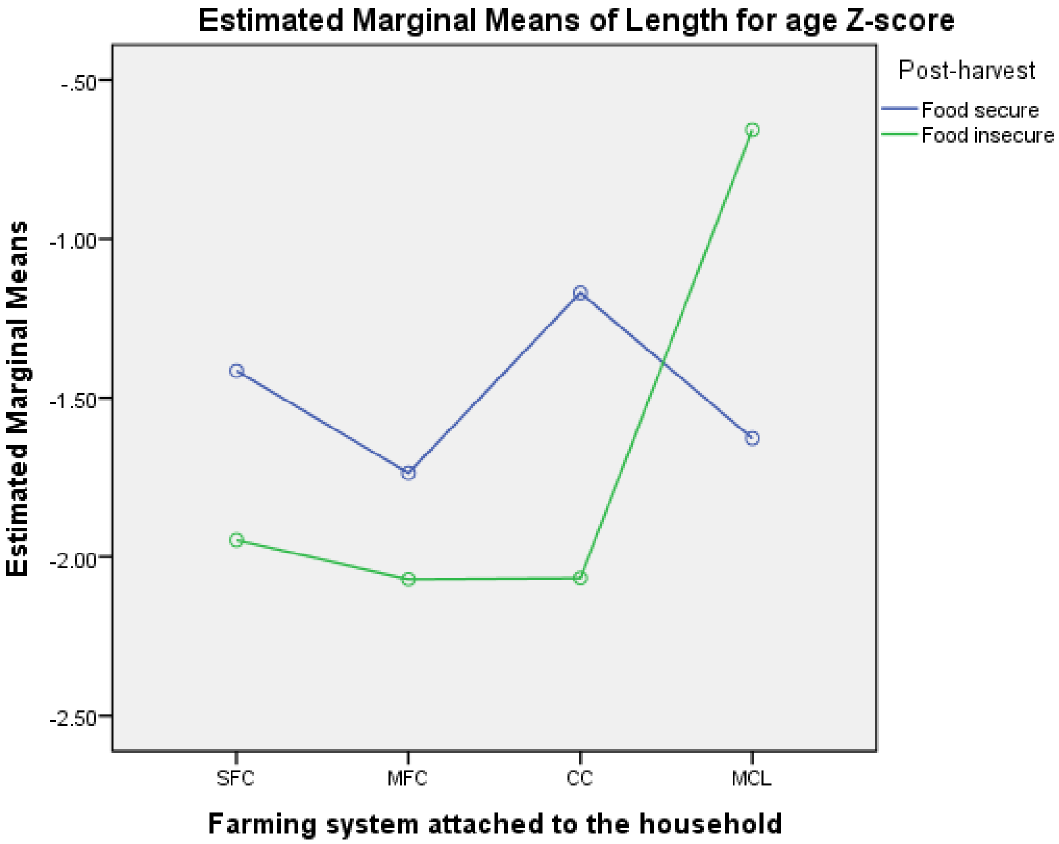

Covariates appearing in the model are evaluated at the following values: How is old is the child?age in month = 10.64, What is your child sex? = 1.48
